# Effects of Between- and Within-Subject Variability on Autonomic Cardiorespiratory Activity during Sleep and Their Limitations on Sleep Staging: A Multilevel Analysis

**DOI:** 10.1155/2015/583620

**Published:** 2015-08-20

**Authors:** Xi Long, Reinder Haakma, Tim R. M. Leufkens, Pedro Fonseca, Ronald M. Aarts

**Affiliations:** ^1^Department of Personal Health, Philips Research, 5656 AE Eindhoven, Netherlands; ^2^Department of Electrical Engineering, Eindhoven University of Technology, 5600 MB Eindhoven, Netherlands; ^3^Department of Behavior, Cognition & Perception, Philips Research, 5656 AE Eindhoven, Netherlands

## Abstract

Autonomic cardiorespiratory activity changes across sleep stages. However, it is unknown to what extent it is affected by between- and within-subject variability during sleep. As it is hypothesized that the variability is caused by differences in subject demographics (age, gender, and body mass index), time, and physiology, we quantified these effects and investigated how they limit reliable cardiorespiratory-based sleep staging. Six representative parameters obtained from 165 overnight heartbeat and respiration recordings were analyzed. Multilevel models were used to evaluate the effects evoked by differences in sleep stages, demographics, time, and physiology between and within subjects. Results show that the between- and within-subject effects were found to be significant for each parameter. When adjusted by sleep stages, the effects in physiology between and within subjects explained more than 80% of total variance but the time and demographic effects explained less. If these effects are corrected, profound improvements in sleep staging can be observed. These results indicate that the differences in subject demographics, time, and physiology present significant effects on cardiorespiratory activity during sleep. The primary effects come from the physiological variability between and within subjects, markedly limiting the sleep staging performance. Efforts to diminish these effects will be the main challenge.

## 1. Introduction

Polysomnography (PSG) is the gold standard and common practice for the objective analyses of overnight sleep architecture (displayed by a so-called hypnogram) and sleep-related disorders such as insomnia/parasomnia, sleep-disordered breathing, and rapid-eye-movement (REM) sleep behavior disorder [1]. With PSG, sleep stages are manually scored on continuous 30 s epochs based on electrophysiological signals including electroencephalogram (EEG), electromyogram (EMG), and electrooculogram (EOG) according to the Rechtschaffen and Kales (R&K) rules [2] or the more recent guidelines of the American Academy of Sleep Medicine (AASM) [3]. PSG recordings are usually acquired in a sleep laboratory that requires a lot of manual labor for visual scoring. It is costly and uncomfortable for subjects and therefore not suited for long-term monitoring. These disadvantages motivated sleep researchers and clinicians to devote more attention to alternatives such as cardiac and respiratory activities, allowing for unobtrusive sleep staging with minimal discomfort to subjects [4–8].

Cardiorespiratory activity has been proven to associate with the autonomic sympathetic and parasympathetic (or vagal) nervous systems in humans, which relates to sleep stages [9–13]. For example, the sympathetic activation of the heart usually translates to an increased spectral power of heart rate variability (HRV) in the low-frequency band between 0.04 and 0.15 Hz and the vagal activity (primarily caused by respiratory sinus arrhythmia) is associated with the spectral power in the high-frequency band between 0.15 and 0.4 Hz [14]. During REM sleep, the high-frequency spectral power increases while the low-frequency spectral power decreases, when compared with non-REM (NREM) sleep and wakefulness [15]. Furthermore, the respiratory volume and frequency are more regular during NREM sleep than during REM sleep and wakefulness [9]. Irregular respiration patterns occurring during wakefulness are usually caused by body movements or alternation of ventilation control manipulated by some external factors; during REM sleep they can be related to muscle atonia or subcortical structures with a possible involvement of the bizarre content of dreams [16, 17].

In addition to sleep stages, the cardiorespiratory activity can be influenced by between-subject variability with respect to (1) subject demographics such as age, gender, and body mass index (BMI) [18–20] and (2) internal physiology such as response of autonomic regulation, metabolic function, and subcortical arousals [21–23]. Note that, for simplicity, here we consider BMI as a demographic. Other factors, which differ from subject to subject and within subjects, such as conscious breathing control and external sleep environment (e.g., noise and temperature), can also cause variations in autonomic response during sleep [24–27]. Furthermore, the autonomic activity appears to change during the course of the night as a function of time and the ratio of NREM and REM sleep in a sleep cycle [13, 28]. These changes would also be reflected in changes of cardiorespiratory activity throughout the night within subjects. Additionally, the daytime activity and any stressful events may change the sleep architecture and consequently affect autonomic control of cardiorespiratory activity during the night [29–31]. It is however not clear to which extent each of these effects can explain the variations in cardiorespiratory activity during sleep.

In regard to automatic sleep staging with autonomic cardiorespiratory activity, parameters are usually derived from cardiac and respiratory signals on a 30 s epoch basis [2, 3]. Due to the existence of between-subject (i.e., interindividual) and within-subject (i.e., intraindividual) variability effects, the correct identification of sleep stages based on the cardiorespiratory parameters seems challenging, in particular when a subject-independent model is used (i.e., when a model is derived from a set of subjects and used to identify sleep stages for other new subjects).

The aim of this fundamental study was to quantitatively investigate the effects of between- and within-subject variability on cardiorespiratory activity during sleep and to evaluate how they are limited to reliable cardiorespiratory-based sleep staging results.

## 2. Materials and Methods

### 2.1. Subjects and Protocol

A total of 165 healthy subjects participating in the SIESTA project [32] were included in this study. The subjects were monitored over a period of three years from 1997 to 2000 in seven different sleep laboratories located in five European countries. The subject demographics (mean ± standard deviation (SD)) including age, gender, and BMI are given in Table 1. The protocol was approved by local ethics committees of all sleep laboratories involved and all subjects provided a written informed consent. The subjects fulfilled the following criteria: no significant medical disorders, no reported symptoms of neurological, mental, medical, or cardiovascular disorders, no history of drug abuse or habituation (including alcohol), no psychoactive medication or other drugs (e.g., beta blockers), no shift work, and usually retirement to bed between 22:00 and 24:00 depending on their habitual bedtime [32].

### 2.2. PSG Recordings

For each subject, single-night full PSG recordings were obtained. Each recording consists of at least 16 channels including EEG (C3-M2, C4-M1, O1-M2, O2-M1, Fp1-M2, and Fp2-M1), EMG (chin and leg), EOG (2 leads), electrocardiogram (ECG, single-channel, modified V1 lead), nasal airflow, respiratory effort (abdominal and chest wall with respiratory inductance plethysmography), snoring (microphone), and blood oxygen saturation [32]. Only the ECG signals, sampled at 100 Hz, 200 Hz, or 256 Hz depending on the equipment setup of each sleep laboratory, and the respiratory (chest) effort signals, all sampled at 10 Hz, were used in this study.

Each PSG recording was visually annotated in 30 s epochs as nighttime wake, REM sleep, and one of the NREM sleep stages S1–S4 by two independent raters according to the R&K rules. In case of disagreement, the consensus annotations between the two raters were obtained. For the analysis in this study, we considered four stages: wake, REM sleep, light sleep (merging S1 and S2), and deep sleep or slow wave sleep (merging S3 and S4). Table 1 presents some sleep statistics of the recording nights.

### 2.3. Data Preparation

The ECG and respiratory effort signals of all subjects were preprocessed before computing the parameters used for analyses. The baseline wander of the ECG signal was removed with a linear phase high-pass filter using an 1.106 s Kaiser window with a 0.8 Hz cutoff frequency and a 30 dB side-lobe attenuation [33]. The resulting signal was normalized with regard to mean and amplitude and a low-complexity precise QRS complex localization algorithm [34] was used to locate the R peaks in the signal. The resulting heartbeat or RR intervals were resampled at 4 Hz using a linear interpolator. To compute the cardiac parameters in the frequency domain, the power spectral density (PSD) of the resampled RR intervals was estimated with an autoregressive model, where the order was adaptive and automatically determined by the Akaike's information criterion (AIC) and was limited to 15 [35]. Using the AR model instead of a Fourier transform was because the Fourier-based approaches may have limitations such as poor spectral resolution and leakage [36], which would be sensitive to estimating the PSD of the RR interval series having a relatively low sampling rate. After that, the spectral power in the low-frequency band and the high-frequency band can be calculated. Note that ectopic RR intervals longer than 2 s, shorter than 0.3 s, or shorter than 0.6 times their previous value were discarded. The epochs were treated as being “invalid” or missing if the coverage was less than 50% (i.e., the sum of the detected RR intervals within an epoch was less than half of the epoch length) since the PSD for these epochs with too many missing heartbeats (likely caused by body motion artifacts) could not be reliably estimated.

The respiratory effort signal was first low-pass-filtered using a 10th order Butterworth filter with a cut-off frequency of 0.6 Hz to eliminate high-frequency noise. Afterwards, the signal baseline was removed by subtracting the median peak-to-trough amplitude estimated over the entire signal. The respiratory peaks and troughs were detected by locating the signal turning points based on sign changes of signal slopes. Finally, we excluded incorrectly detected peaks and troughs (1) in peak-to-trough or trough-to-peak intervals where the sum of two successive intervals was less than the median of all intervals over the entire recording and (2) with amplitudes where the peak-to-trough difference was smaller than 0.15 times the median of the entire-night respiratory signal [37].

### 2.4. Cardiorespiratory Parameters

We analyzed six cardiorespiratory (two respiratory and four cardiac) parameters. The respiratory parameters were the mean breathing rate or respiratory frequency (BR) and the standard deviation of breathing rates (SDBR). For cardiac activity, the time-domain parameters included the mean heart rate (HR) and the standard deviation of heartbeat intervals (SDNN). The spectral-domain parameters included the spectral power of heartbeat intervals in the low-frequency band (LF) and the spectral power in the high-frequency band (HF). The LF and HF were normalized by dividing them by the total spectral power minus the power in the very-low-frequency (VLF, 0.003–0.04 Hz) band [14, 38]. This resulted in their expressions in a normalized unit (nu) instead of the absolute unit (ms^2^). The normalization can minimize the effect on the LF and HF values caused by the changes in total spectral power. And the normalized LF and HF represent the relative power in each frequency band in proportion to the total power minus VLF power, emphasizing the controlled and balanced behavior of the two aspects (i.e., sympathetic and parasympathetic activities) of the autonomic nervous system [14].

All the six parameters have been widely used for the task of cardiorespiratory-based sleep staging [6, 37, 39–41]. A logarithmic transformation was applied to BR, SDBR, HR, and SDNN to correct for nonsymmetry in the frequency distributions. Measurement units are therefore expressed in natural logarithmic Hz (ln-Hz) for BR and SDBR, natural logarithmic beats per minute (ln-bpm) for HR, and natural logarithmic millisecond (ln-ms) for SDNN.

### 2.5. Descriptive Statistics

Values of the cardiorespiratory parameters (mean ± SD) measured from subjects with different demographics (gender, age, and BMI) and time of night are presented. We considered different cohort sets including three age groups: young (20–39 y), middle (40–69 y), and elderly (>69 y), and three BMI groups: underweight (<18.5 kg·m^−2^), normal weight (18.5–25 kg·m^−2^), and overweight (>25 kg·m^−2^). In addition, total sleep time was divided into four periods: 0–90 min, 90–180 min, 180–270 min, and >270 min. Significance of difference between groups was tested with the analysis of variance (ANOVA)* F* test.

### 2.6. Multilevel Analysis

Traditional statistical methods such as repeated measures ANOVA (rANOVA), repeated measures multivariate ANOVA (rMANOVA), and multiple regression analysis (MRA) are often used to analyze longitudinal data. However, they might not be appropriate since they expect uncorrelated and independent observations or they cannot model variables in different levels [42]. In regard to the nature of multiple dependent variables, a more generalized multilevel (regression) analysis [43] takes structural variables with fixed and random effects measured at multiple hierarchical levels into account. Compared with the traditional methods, multilevel analysis has several advantages [43, 44]. First, it serves to deal with incomplete data while ANOVA-based methods handle that by simply deleting all subjects with missing measures. Second, it concerns data with a hierarchical structure and thus allows for meta-analysis of explanatory variables with effects on different levels simultaneously while MRA usually considers variables at the same level. Third, it is able to quantify the variability explained only within levels. To these matters, we applied multilevel models to statistically evaluate the effects of between- and within-subject variability on the cardiorespiratory parameters. Under a variety of names used by different authors, multilevel models are also known as mixed models, random effects models, and hierarchical linear models [43].

Due to the presence of its advantages, multilevel analysis has been widely deployed in many areas such as psychophysiology [42], sociology [45], biology [46], and medicine [47]. In the field of sleep study researchers have applied multilevel models for investigating daily associations (within-subject and daily variability) between sleep and effect [48]; stress-dependent within-subject variability in sleep duration and sleep fragmentation [49]; age and between-subject variability in reaction time performance with sleep restriction [50]; relationship between self-reported and PSG-measured sleep times [51]; between-subject variability in “sleep need” and “vulnerability to sleep loss” [52]; circadian variation of cardiac autonomic activity [53]; and habitual traffic noise effect on respiratory sinus arrhythmia during sleep [54]. To the authors' knowledge, analyzing between- and within-subject effects on cardiorespiratory activity during sleep (across sleep stages) based on multilevel models has not been studied.

#### 2.6.1. Between- and within-Subject Effects

On the one hand the between-subject variability effects of cardiorespiratory activity can be linked to physiology and subject demographics (age, gender, and BMI). On the other hand, cardiorespiratory activity may change depending on the time of night within subjects [13]. This time effect can also vary between subjects. Most multilevel models assume homogeneity or equality of variance for each prediction variable, whereas this might not hold for the time effect. Therefore, it is hypothesized that the time effect also changes along with subject demographics. This can be evaluated by “cross-interactions” between time and demographic variables. Here we did not take into account the influences from the differences in sleep environment, daytime energy expenditure, and other factors or behaviors such as stress, smoking, and personality. These influences, if existent, were assumed to be conveyed by the physiological variability. Additionally, in our previous work [55], there were no effects on the cardiac activity found between different laboratories based on the same data. For this reason, we disregarded the laboratory factor during our modeling procedure.

To evaluate the between- and within-subject effects, we constructed a multilevel model with two levels (level two: subject; level one: time or epoch) for a given cardiorespiratory parameter *y*. The model predicts/estimates the values of the parameter based on a set of variables including sleep stages, age, gender, BMI, and time of night. For the parameter value *y*_*ij*_ in the *i*th epoch of the night (*i* = 1,2,…, *N* with a total of *N* epochs) from subject *j*  (*j* = 1,2,…, *M* where *M* is the total number of subjects), the two-level regression model with associated coefficients is given by

  Model #1:
  (1) yij=β0+μ0j+∑sβs+μsjsij+βt+μtjtimeij+e0ij+βaagej+βggenderj+βbBMIj+βtatime×ageij+βtgtime×genderij+βtbtime×BMIijwith  μ0jμsjμtj~N000,Ω0ΩsΩt,  e0ij~N0,Ωe,

in which *β*_0_ is the fixed intercept, *μ*_0*j*_ is the random effect with variance *Ω*_0_ indicating the between-subject variability in physiology (independent of sleep stages or corrected by sleep stages), and *e*_0*ij*_ is the (random) residual term with variance *Ω*_*e*_ quantifying the within-subject physiological variability (independent of time). *s* represents sleep stages (*s* = wake, REM sleep, light sleep, and deep sleep), where wake, REM sleep, light sleep, and deep sleep are all dummy/binary variables (1 or 0 indicating “yes” or “no”). This means that the multinomial sleep stage information is expressed by the sum of the four dummy sleep stage variables where only one is nonzero (=1) for each epoch. Hence, the term ∑_*s*_(*β*_*s*_ + *μ*_*sj*_)*s*_*ij*_ specifies the sleep stage of epoch *i* from subject *j* with its fixed effect *β*_*s*_ and random effect *μ*_*sj*_, where *Ω*_*s*_ reflects the between-subject physiological variability in sleep stage *s*. The demographic variables age (y), gender (dummy variable: 0 = man and 1 = woman), and BMI (kg·m^−2^), respectively, correspond to the fixed effects *β*_*a*_, *β*_*g*_, and *β*_*b*_ varying between subjects. The variable time_*ij*_ (min) expresses the relative time of epoch *i* (time_*ij*_ = *i*/2) from subject *j*, *β*_*t*_ is the fixed time effect corresponding to linear changes over time within subjects, *μ*_*tj*_ is the random time effect with variance *Ω*_*t*_ indicating the variability of time effect between subjects, and *β*_*ta*_, *β*_*tg*_, and *β*_*tb*_ are cross-interactions specifying the fixed age-, gender-, and BMI-related time effects, respectively. Note that the variances from the random effects (including residuals) were assumed to be drawn from a normal distribution with zero mean. Here the normality was visually checked using a heuristic Quantile-Quantile (Q-Q) plot method since the commonly used numerical normality tests are not appropriate on large-sized samples [56].

#### 2.6.2. Centering Effect

Intuitively, the mean value of a specific cardiorespiratory parameter over the entire night may differ from subject to subject, which might be due to the physiological variability between subjects at the general mean level. Cronbach [57] proposed a model that regards an additional predictor indicating the between-group centering effect in real applications, allowing for expressions of parameter values as deviations from the group means. In this study, the model with centering (physiological) effect for a given parameter can be expressed as

  Model #2:
  (2) yij=β0+μ0j+∑sβs+μsjsij+βt+μtjtimeij+βcy−j+e0ij+βaagej+βggenderj+βbBMIj+βtatime×ageij+βtgtime×genderij+βtbtime×BMIijwith  μ0jμsjμtj~N000,Ω0ΩsΩt,  e0ij~N0,Ωe,

where ${\overset{-}{y}}_{j}$ is the variable that gives the within-subject mean value over the entire night for subject*j* and its associated fixed slope *β*_*c*_ corresponds to the between-subject centering effect. This effect is meant to reflect the physiological difference between subjects at the (individual) overnight mean level. Here the estimation of the overnight mean value was assumed to be independent of sleep stage composition (percentages of sleep stages) over the entire night. To a certain degree, the demographic effects were expected to be conveyed by the centering effect. Therefore, the model without the centering term (Model #1) should be used for exploring the actual demographic effects with a single model.

#### 2.6.3. Model Estimation and Optimization

The multilevel modeling was implemented using the MLwiN software (Centre for Multilevel Modeling, the University of Bristol, UK), where an iterated generalized least square (IGLS) algorithm is issued for the model estimation, that is, the estimates of regression coefficients and their variances [58]. The model goodness-of-fit can be evaluated by the deviance (measured by −2·log-likelihood) obtained during the modeling procedure.

A Wald* Z*-test was used to statistically examine the significance of the effects, testing the null hypothesis that a coefficient equals zero [43]. For each estimated model coefficient or variance *γ* corresponding to a specific effect, the Wald* Z* statistic is computed as the square of the estimated coefficient divided by its standard error (SE):

(3) $$\begin{array}{c} Z=\frac{\gamma^{2}}{{SE}^{2}\left(\gamma \right)}. \end{array}$$

The acceptance or rejection of the null hypothesis can be tested with a Chi-squared (*χ* ^2^) test with one degree of freedom (df).

The models described in (1) and (2) are “full” models and need to be optimized by excluding the effects with coefficients statistically not different from zero (tested with the Wald statistic). Differences between models are assessed by comparing model deviances using a *χ* ^2^ statistic (i.e., likelihood ratio test) with df = 2. This paper only presents the results of the optimized models that are manipulated by significant effects.

### 2.7. Explanations of Variance

It is of particular interest in interpreting how much the model variance is explained by different variables or effects. As described in Table 2, a total of seven explanatory effects for each cardiorespiratory parameter were considered in this study. Raudenbush and Bryk [59] proposed an approach by using the squared multiple correlation *R* ^2^ to derive the proportion of variance modeled by means of explanatory variables with corresponding effects (proportion of variance explained, PVE). This approach examines the residual variances in a sequence of models. Suppose that the full model under consideration for a given parameter is Model #2, given by (2). A sequence of seven models (Models A–G) can be established in a certain order that serves to compute the PVE of each effect. The detailed procedure of doing this is described in the Appendix.

### 2.8. Between- and Within-Subject Effects in Sleep Staging

#### 2.8.1. Sleep Staging Algorithm

Linear discriminant (LD) has been shown to be an appropriate algorithm in classifying overnight sleep stages based on cardiorespiratory activity in many studies [6, 41]. In this work we adopted an LD classifier to perform automatic sleep staging. Overall accuracy and the Cohen's Kappa coefficient of agreement [60] were used to evaluate the classifier's performance. Additionally, sleep statistics including the percentages of wake, REM sleep, light sleep, and deep sleep were calculated. In order to verify the classification performance, the subjects were randomly divided into a set of 82 subjects used to train the classifier and a set of the other 83 subjects for testing.

#### 2.8.2. Comparison of Correction Schemes

The objective was to examine how much the between- and within-subject effects on the cardiorespiratory activity would restrict the performance in classifying sleep stages (wake, REM sleep, light sleep, and deep sleep) and then estimating the sleep statistics. For comparison, we analyzed three different “correction” schemes (CS) based on the optimized Model #2 with estimated model coefficients to correct (or predict) the values for each parameter. The corrected values were then used to perform sleep staging. The sleep staging using the original measured values without any corrections served as the baseline scheme (BS).

(i) The first correction scheme (CS1) predicts the parameter values with subtraction of all the fixed effects independent of sleep stages, such that
 CS1:
  (4) $$\begin{array}{c} {\hat{y}}_{ij}=\mu_{0j}+\underset{s}{\sum }\left(\;\beta_{s}+\mu_{sj}\;\right)s_{ij}+\mu_{tj}{\text{time}}_{ij}+e_{0ij}. \end{array}$$
(ii) The second correction scheme (CS2) corrects the parameter values by subtracting all the (sleep stage independent) fixed effects and all the between-subject random effects, such that
 CS2:
  (5) $$\begin{array}{c} {\hat{y}}_{ij}=\underset{s}{\sum }\beta_{s}s_{ij}+e_{0ij}. \end{array}$$
(iii) The third correction scheme (CS3) excludes all the (sleep stage independent) fixed effects and the within-subject effect to correct the parameter values, such that
 CS3:
  (6) $$\begin{array}{c} {\hat{y}}_{ij}=\mu_{0j}+\underset{s}{\sum }\left(\;\beta_{s}+\mu_{sj}\;\right)s_{ij}+\mu_{tj}{\text{time}}_{ij}. \end{array}$$

Note that, again, the exclusive aim of analyzing these correction schemes in the present study was to evaluate in what aspect and how far the cardiorespiratory parameters can be improved for sleep staging instead of really performing sleep staging. In other words, we intended to answer the question, what sleep staging performance can be achieved if we can eliminate the effects caused by the between- or within-subjects variability? Investigating methods of estimating the fixed coefficients and random variances without knowing sleep stages was not addressed in this study.

## 3. Results

### 3.1. Descriptive Results

**Figure 1:**
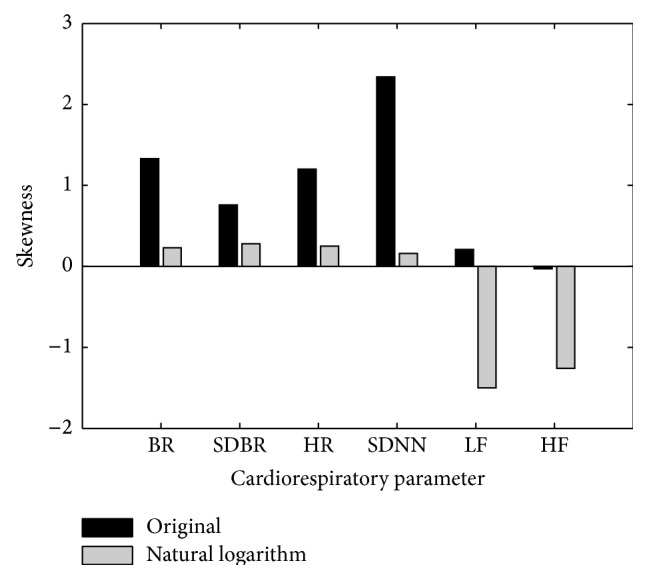
Skewness comparison of cardiorespiratory parameters with and without natural logarithm transformation, indicating that BR, SDBR, HR, and SDNN should be log-transformed.

Figure 1 compares the skewness of the parameters with and without transformation using natural logarithms. It indicates that the four parameters BR, SDBR, HR, and SDNN need to be log-transformed since they were of skewed distribution and their skewness values largely decreased after performing the log-transformation.  Table 3 shows the values (mean ± SD) of the six cardiorespiratory parameters BR, SDBR, HR, SDNN, LF, and HF analyzed in this study for different cohort sets in different genders, age groups, BMI groups, time periods, and sleep stages. The values significantly differed across different groups for all the cohort sets (ANOVA* F*-test,* P* < 0.001).

### 3.2. Multilevel Modeling

In comparison with the* F*-test, the multilevel regression models enable a more adequate and thorough statistical analysis. With the multilevel Model #1, the estimated coefficients and variances for all the parameters are shown in Table 4. As a result of removing the insignificant variables (tested using the Wald* Z*-test with* P* > 0.05) except for the constant intercept and sleep stage variables, the model was optimized. The table indicates that the demographics significantly influenced the cardiorespiratory activity from different aspects. Upon a closer look, it is found that the breathing rate, BR, for the healthy subjects with a higher BMI was significantly higher than the subjects with a lower BMI (0.011 ln-Hz per kg·m^−2^,* P* < 0.01) at the baseline of −1.458 ln-Hz, whereas its variation SDBR remained the same. For cardiac activity, the mean heart rate HR of women was higher than men (0.042 ln-bpm,* P* < 0.05) at the baseline of 4.221 ln-bpm while its variation SDNN were lower than men (−0.247 ln-ms,* P* < 0.0001) at the baseline of 4.823 ln-ms. SDNN were also negatively correlated to subject age (−0.009 ln-ms per y,* P* < 0.0001) and BMI (−0.025 ln-ms per kg·m^−2^,* P* < 0.01). With the spectral analysis of HRV, men had an LF power increased by 0.045 nu (*P* < 0.05) but a lower HF power of 0.052 nu (*P* < 0.01) compared with women during bedtime sleep. The HF power slightly decreased along with the increase in age (−0.002 nu per y,* P* < 0.05). These results are consistent with previous work [18, 61, 62].

**Figure 2:**
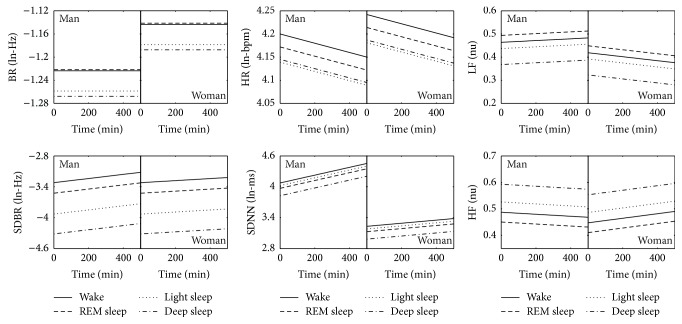
Anexample of multilevel regressions of the six cardiorespiratory parameters for a man (age: 24 y, BMI: 21.3 kg·m^−2^) and a woman (age: 70 y, BMI: 28.6 kg·m^−2^) using coefficients estimated through Model #1 excluding the random coefficients and residual term. The regression variables included age, gender, BMI, time, and time × age, time × gender, time × BMI, and sleep stages: wake, REM sleep, light sleep, and deep sleep.

Most of the analyzed parameters were found to be time-variant (i.e., they were modulated by time of night) with an exception of breathing rate (Table 4). For instance, the heart rate HR dropped down gradually along with the time progression over the night (−0.0001 ln-bpm per min,* P* < 0.0001) at the baseline of 4.221 ln-bpm while the variation in heartbeat intervals SDNN increased (0.001 ln-ms per min,* P* < 0.0001) at the baseline of 4.823 ln-ms, confirming the findings reported previously [63]. This time modulation varied from subject to subject because of the presence of significant variance *Ω*_*t*_ (*P* < 0.0001), referring to the random time effect. The time was also modulated by some demographic variables (such as age for SDNN and BMI for SDBR, LF, and HF). We note in the table that there appeared to be significant between-subject physiological effects for all parameters (*P* < 0.0001), measured by the random variances of sleep stage variables. These variances seemed approximately homogeneous across sleep stages for BR and HR but were clearly different for their variations SDBR and SDNN.  Figure 2 illustrates an example that compares the parameter values (estimated by multilevel regression based on Model #1) changing along with time between two subjects with different demographics. It shows that the fixed time and demographic effects were generally larger than the differences between sleep stages.

With the addition of the centering variable to Model #1, we have Model #2 and the estimated regression coefficients after model optimization (Wald* Z*-test at* P* < 0.05, for each coefficient) are shown in Table 5. As stated, this model included the between-subject physiological effect at the overnight mean level (i.e., centering effect), resulting in an obvious reduction of the random variance in each sleep stage compared with Model #1. This indicates that regardless of sleep stage the between-subject variability in physiology can be reflected, to a certain degree, by the difference of the mean value over night. Besides, centering the parameter values per subject slightly influenced the time effect in both fixed and random parts. In comparison with Model #1, a lower deviance using Model #2 was obtained for all the parameters (*P* < 0.0001) as shown in Tables 4 and 5, indicating a better goodness-of-fit on the parameters using the model with the centering variable.

**Figure 3:**
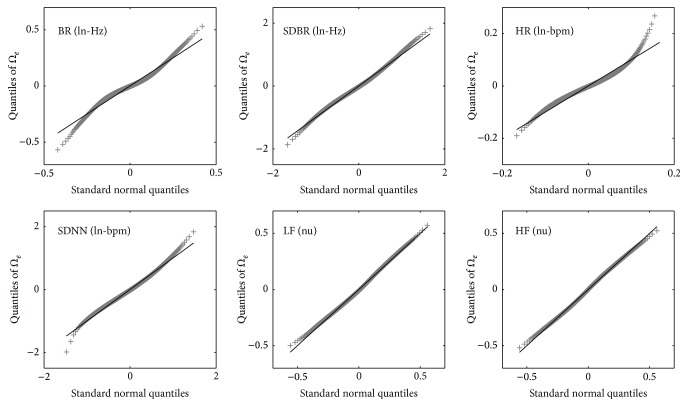
Q-Q plots of residual variance *Ω*_*e*_ of the multilevel models (Model #1) for the six cardiorespiratory parameters. These plots suggest approximate normal distributions of the residual variances.

Normality of the variances was tested and suggested using the Q-Q plot method for all models. For example, the Q-Q plots of the residual variances *Ω*_*e*_ (in Model #1) for all the parameters are shown in Figure 3, suggesting that the variances were approximately drawn from a normal distribution.

### 3.3. Proportion of Variance Explained (PVE)

To discover which effects explained the variance and how much each constituted we computed for each cardiorespiratory parameter the PVE for each effect by analyzing the estimated variances of random intercept and residual in a sequence of models (Models A–G in the Appendix). The variance changes in the models with the inclusion of different effects in a specific order are shown in Table 6, based on which the PVE values were obtained in Table 7. Note that the variances explained by sleep stages were not included in PVE. For BR and HR, the between-subject centering effects dominated the variances (55.26% for BR and 77.95% for HR), indicating that the subjects behaved differently with respect to their breathing rate and heart rate at the general mean level throughout the whole night. We also see that the variations in breathing rate and heart rate had a lower centering difference between subjects (with PVE of 26.23% for SDBR and of 39.06% for SDNN) compared with the physiological variability within subjects (with PVE of 61.69% for SDBR and of 40.87% for SDNN). This was also the case for LF and HF powers in the spectral domain of HRV as shown in Table 7. As a result, the overall between-subject variability had more influence on breathing rate (PVE of 66.58%) and heart rate (PVE of 86.25%) while less on their variations (PVE of 37.94%, 58.66%, 33.62%, and 35.13% for SDBR, SDNN, LF, and HF, resp.) compared with the overall within-subject variability. In general, the variances explained by the effects in physiology between subjects (including the effect at the overnight mean level and random effect) and within subjects accounted for 83.83–97.16% of the total variance for different cardiorespiratory parameters.

Specifically, a relative larger percentage (13.7%) of the demographic effect can be found on SDNN compared with the other parameters. The PVE of between-subject physiological variability (in the random part) ranged from 2.27% to 7.62% depending on the parameters. For the time effect, the PVE in the fixed part (0.01–1.32%) reflecting the linear changes of parameters over time within subjects was smaller than in the random part (1.58–2.74%) with the indication of different changes over time between subjects. In general, the time effect accounted for much less of the total variance than most other effects. Finally, although the cross-interactions existed between time and demographics for BR, SDNN, LF, and HF, the proportion of variance they explained was very small (<0.20%).

### 3.4. Sleep Staging Results

The results of sleep staging are presented in Table 8, where different schemes (BS and CS1–CS3) were compared. We observe that the correction by means of the between- and/or within-subject effects for the parameters generally enabled performance improvement in sleep staging (by comparing the results of CS1–CS3 with BS). In particular, correcting the parameters by the fixed effects (demographics, time, and their cross-interactions) independent of sleep stages (CS1) resulted in a significantly increased Kappa of 0.29 ± 0.11 and a significantly increased accuracy of 60.4 ± 8.8% (Wilcoxon test,* P* < 0.00001) compared with the baseline without any correction (Kappa of 0.19 ± 0.10 and accuracy of 55.8 ± 9.8%). In addition, if we further correct the variability of the parameters evoked by the between-subject random effects (CS2), the sleep staging results significantly increase to a Kappa of 0.35 ± 0.09 and an accuracy of 62.9 ± 7.8% (Wilcoxon test,* P* < 0.00001), where the SD of results over subjects is simultaneously reduced. On the other hand, if the within-subject variability is corrected (CS3), the sleep staging performance is markedly improved (at a Kappa of 0.72 ± 0.23 and an accuracy of 83.5 ± 14.4%) (Wilcoxon test,* P* < 0.00001), but the SD would increase because this correction scheme focused on reducing effects within subjects rather than those between subjects. Similarly, as shown in Table 8, correcting the parameters could help obtain a more accurate estimation of sleep stage composition.

## 4. Discussion

The results of demographic and time of night effects found in this study are consistent with the findings reported in previous work [18, 61–63]. For example, Brandenberger et al. [18] suggested that, compared with young subjects, older subjects have a marked fall in HRV without sleep stage dependent variation due to the withdrawal in vagal activity (and increased sympathetic activity) generally associated with decreased sleep quality. Also, “periodic breathing” that often interrupts the normal breathing pattern for the elderly is the major trigger for HRV fluctuations through autonomic efferents, which can induce substantial modification in HRV, possibly leading to the unseen age effect in overall breathing rate and its variation. It has been reported that gender affects cardiac dynamics where women have relatively greater HF cardiac fluctuations than men, while this is not apparent for respiratory activity [62]. However, this effect was later found to be sleep stage dependent that can only be observed during wakefulness and REM sleep because the predominant loss in vagal activity is often associated with the disruption of homeostasis for men with increased physiological vulnerability or “stress” [61]. Therefore, a modified multilevel model expressing gender differences relying on sleep stages should be investigated in future work. In addition, the autonomic change (e.g., decreased sympathetic and increased vagal activity) over time throughout the night has been shown to be associated with circadian influences [13, 63] and “sleep pressure” [64]. No significant or weak correlation was found between BMI and autonomic nervous regulation [19, 65].

It should be noted that the model used to facilitate the interpretation of the demographic effects (Model #1) should not include the (between-subject) centering variable. This is because the demographic differences usually correspond to the autonomic changes at the overnight mean level. Due to the inclusion of the centering effect in Model #2, it came as a surprise that some demographic variables still had significant effects (see Table 4), which contradicts our expectation that their effects on the cardiorespiratory activity are fully manifested by the parameter mean values. The cause is that the percentages (or composition) of sleep stages were not exactly the same for all subjects. Therefore, the demographic differences were only partially explained by the centering variable and the unexplained part depends on the difference of sleep stage composition between subjects.

It is important to note that since some effects were correlated with each other, the order in the procedure of constructing the sequence of models (see the Appendix) must be specifically determined. This aimed at precisely quantifying the proportion of variance explained by each effect. The procedure should follow the way that the model with fixed effects (e.g., demographic effects) that are explainable by other effects should be first addressed and the model with random effects should be included later [43].

In Tables 4 and 5, it can be seen that the time variable was able to explain variance at the subject level due to the significance of the random time effect. First, the slope of cardiorespiratory activity changing over time might depend on sleep stages (or their transitions) and thus might not be with a continuous linear trend. A method of handling the sleep stage dependency is to use a model that contains the cross-interactions between sleep stages and time; but for the influence of sleep stage transitions, it is suggested to regard the night as different segments without any sleep stage transitions. Second, the random time effect could likely be due to the difference in autonomic control or changes in sleep architecture between subjects by other factors such as daytime activity, work stress, and response to the sleep environments during sleep. This was not addressed in this study and it merits further investigation. On the other hand, the cross-interactions between time and demographics (in particular, BMI) explained some total variance at both subject and epoch levels. Although the amount and proportion of variance explained by the time-related effects seem much smaller than some other effects as shown in Table 7, they are still statistically unequal for different subjects and are relatively large compared with the differences between sleep stages for some parameters such as LF and HF, especially at the end of the night, which can be observed in Figure 2.

Regarding the quantified between- and within-subject effects, they were found to be statistically significant and they explained a relatively large portion of the total variance as we expected. In fact, several factors in addition to internal physiology may also explain some of the total variance within subjects in cardiorespiratory activity such as body movements, body position, sleep environment, conscious breathing control, and even daytime activity. However, we did not answer which of these effects takes place in this work and this should be studied in the future.

When evaluating the performance of sleep staging using the cardiorespiratory parameters, Model #2 should be regarded as the preference. For each parameter, although the estimate of its overnight mean value for each subject was not completely accurate (due to the difference of sleep stage composition between subjects), correcting it can still result in a reduction of the physiological variability between subjects to a great extent. As a consequence, the sleep staging results can be improved. Table 6 confirms that the centering effect actually constituted a large proportion of the total variance. Moreover, Figure 2 illustrates that the variations of the parameters caused by demographic and time effects were somewhat comparable with or even larger than the differences between sleep stages, leading to difficulty in separating sleep stages. With respect to the capability of the parameters in classifying sleep stages, Table 5 shows that, for example, SDBR had a larger difference between sleep stages compared with the other parameters while BR had no difference between REM sleep and wakefulness. This indicates that the intrinsic separation of sleep stages should vary between the parameters that express different aspects of the autonomic activity.

Table 8 indicates that the between- and within-subject variability conveyed by the cardiorespiratory activity limited the sleep staging performance. To improve it, the correction scheme CS1 seems potentially applicable from a practical point of view because the fixed effects are usually prior information that is independent of sleep stages or they can be estimated from the training data before performing sleep staging. However, realizing CS2 and CS3 requires either information of sleep stages (which appear practically unknown and need to be identified) or estimation of random variances (which are hardly predictable for new subjects). Therefore, the challenge will be on how to diminish the random effects caused by either between-subject variability or within-subject variability when sleep stages are unknown. For instance, normalizing the parameter values based on their variation or distribution throughout the night for each subject might allow for reduction of the between-subject random effect in physiology to some extent. Incorporating more explanatory variables in the model that are independent of sleep stages and are able to explain some variance of the model would help better correct the parameters. Compared to the parameters analyzed in this study, exploring new parameters with smaller random variances (i.e., those that are less influenced by the between- or within-subject physiological variability) or additional information in separating sleep stages may improve the sleep staging variability performance. Nevertheless, we argue that the performance of cardiorespiratory-based sleep staging will always be limited unless the between- and/or within-subject random variances are successfully explained and corrected.

## Figures and Tables

**Table 1:** Subject demographics and sleep statistics (*n* = 165).

	Mean ± SD	Range
Gender (77 men and 88 women)		
Age, y	51.8 ± 19.4	20–95
BMI, kg·m^−2^	24.6 ± 3.5	17.0–35.3
Total recording time, h	7.8 ± 0.5	6.0–9.3
Wake, %	22.7 ± 13.2	1.2–78.6
REM sleep, %	13.6 ± 5.3	0–26.5
Light sleep, %	52.3 ± 10.0	15.6–72.1
Deep sleep, %	11.4 ± 6.6	0–28.5

**Table 2:** Description of the seven explanatory effects (with exclusion of sleep stage effects) on cardiorespiratory activity considered in this study.

Effect	Description
Overall between-subject effect	
Demographic effect	Fixed effect, variability in age, gender, and BMI between subjects
Centering (physiological) effect	Fixed effect, variability in overnight mean level between subjects
Between-subject time effect	Random effect, variability in time of night between subjects
Between-subject physiological effect	Random effect, variability in physiology between subjects
Overall within-subject effect	
Within-subject time effect	Fixed effect, variability in time of night within subjects
Within-subject physiological effect	Random effect, variability in physiology within subjects
Cross-interaction effect	
Demographic-related time effect	Fixed effect, demographic-related variability in time of night

**Table 3:** Values (mean ± SD) of the six cardiorespiratory parameters in different cohort sets.

Cohort set	Respiratory parameters		Cardiac parameters			
(*n* = 165)	BR, ln-Hz	SDBR, ln-Hz	HR, ln-bpm	SDNN, ln-ms	LF, nu	HF, nu
Gender						
Man	−1.20 ± 0.24	−3.67 ± 0.75	4.13 ± 0.15	3.74 ± 0.77	0.42 ± 0.23	0.47 ± 0.23
Woman	−1.22 ± 0.23	−3.81 ± 0.76	4.16 ± 0.16	3.49 ± 0.71	0.39 ± 0.22	0.50 ± 0.23

Age						
Young	−1.24 ± 0.24	−3.85 ± 0.74	4.11 ± 0.16	3.94 ± 0.63	0.36 ± 0.20	0.56 ± 0.22
Middle	−1.20 ± 0.24	−3.71 ± 0.78	4.15 ± 0.16	3.52 ± 0.69	0.45 ± 0.23	0.45 ± 0.23
Elderly	−1.18 ± 0.20	−3.70 ± 0.71	4.17 ± 0.13	3.39 ± 0.81	0.38 ± 0.24	0.45 ± 0.22

BMI						
Underweight	−1.24 ± 0.14	−4.00 ± 0.66	4.11 ± 0.12	4.01 ± 0.53	0.36 ± 0.18	0.56 ± 0.19
Normal	−1.23 ± 0.23	−3.77 ± 0.74	4.14 ± 0.16	3.72 ± 0.73	0.41 ± 0.22	0.48 ± 0.23
Overweight	−1.18 ± 0.24	−3.70 ± 0.77	4.15 ± 0.15	3.46 ± 0.75	0.39 ± 0.23	0.48 ± 0.23

Time of night						
0–90 min	−1.22 ± 0.22	−3.81 ± 0.80	4.16 ± 0.15	3.52 ± 0.73	0.39 ± 0.22	0.50 ± 0.23
90–180 min	−1.21 ± 0.22	−3.85 ± 0.75	4.17 ± 0.15	3.58 ± 0.74	0.42 ± 0.23	0.46 ± 0.23
180–270 min	−1.20 ± 0.23	−3.77 ± 0.77	4.15 ± 0.16	3.61 ± 0.77	0.41 ± 0.23	0.48 ± 0.23
>270 min	−1.21 ± 0.24	−3.66 ± 0.72	4.12 ± 0.15	3.67 ± 0.75	0.40 ± 0.22	0.49 ± 0.23

Sleep stage						
Wake	−1.16 ± 0.23	−3.25 ± 0.62	4.19 ± 0.15	3.61 ± 0.78	0.42 ± 0.24	0.44 ± 0.23
REM sleep	−1.18 ± 0.22	−3.44 ± 0.52	4.15 ± 0.16	3.64 ± 0.76	0.45 ± 0.23	0.42 ± 0.22
Light sleep	−1.23 ± 0.23	−3.89 ± 0.73	4.13 ± 0.15	3.64 ± 0.73	0.40 ± 0.22	0.49 ± 0.23
Deep sleep	−1.24 ± 0.23	−4.29 ± 0.71	4.14 ± 0.15	3.45 ± 0.72	0.33 ± 0.21	0.57 ± 0.21

Note: ln, natural logarithm; nu, normalized unit; young, 20–39 y; middle, 40–69 y; elderly, >69 y; underweight, <18.5 kg·m^−2^; normal weight, 18.5–25 kg·m^−2^; overweight, >25 kg·m^−2^; light sleep, S1 and S2 stages; deep sleep, S3 and S4 stages. For all the parameters, values between each cohort group were significantly different (*F*-test, *P* < 0.001) but this may be imprecise since subject demographics, time of night, and sleep stages were possibly not independent.

**Table 4:** Coefficients and their standard errors (SE) of the optimized multilevel model without the between-subject centering effect (Model #1) for the six cardiorespiratory parameters analyzed in this study.

Model coef.	Respiratory parameters		Cardiac parameters			
	BR, ln-Hz	SDBR, ln-Hz	HR, ln-bpm	SDNN, ln-ms	LF, nu	HF, nu
Fixed	Coefficient (SE)					
*β*_0_	−1.458 (0.087)	−3.320 (0.032)	4.221 (0.016)	4.823 (0.255)	0.464 (0.014)	0.535 (0.027)
*β*_wake_	Baseline	Baseline	Baseline	Baseline	Baseline	Baseline
*β*_REM_	0.002 (0.008)^NS^	−0.205 (0.026)	−0.028 (0.001)	−0.104 (0.027)	0.030 (0.007)	−0.037 (0.007)
*β*_light_	−0.035 (0.008)	−0.611 (0.026)	−0.061 (0.001)	−0.052 (0.021)	−0.027 (0.006)	0.039 (0.006)
*β*_deep_	−0.044 (0.010)	−0.997 (0.033)	−0.055 (0.001)	−0.249 (0.026)	−0.096 (0.008)	0.106 (0.008)
*β*_*a*_				−0.009 (0.002)		−0.002 (0.001)
*β*_*g*_			0.042 (0.021)	−0.247 (0.069)	−0.045 (0.018)	0.052 (0.017)
*β*_*b*_	0.011 (0.004)			−0.025 (0.011)		
*β*_*t*_		0.001 (0.0004)	−0.0001 (0.2*e* − 4)	0.001 (0.0002)	0.0004 (0.0001)	−0.0004 (0.0001)
*β*_*ta*_				−1.0*e* − 5 (0.3*e* − 5)		
*β*_*tg*_						
*β*_*tb*_		−2.8*e* − 5 (1.3*e* − 5)			−1.7*e* − 5 (0.5*e* − 5)	1.7*e* − 5 (0.5*e* − 5)

Random	Coefficient (SE)					
Ω_0_						
Ω_wake_	0.030 (0.003)	0.159 (0.018)	0.018 (0.002)	0.224 (0.025)	0.018 (0.002)	0.014 (0.002)
Ω_REM_	0.029 (0.003)	0.171 (0.020)	0.019 (0.002)	0.280 (0.031)	0.022 (0.002)	0.018 (0.002)
Ω_light_	0.030 (0.003)	0.219 (0.024)	0.020 (0.002)	0.256 (0.028)	0.019 (0.002)	0.017 (0.002)
Ω_deep_	0.031 (0.003)	0.257 (0.029)	0.020 (0.002)	0.324 (0.036)	0.020 (0.002)	0.017 (0.002)
*Ω*_*t*_	1.2*e* − 7 (0.1*e* − 7)	6.6*e* − 7 (0.8*e* − 7)	3.5*e* − 8 (0.4*e* − 8)	7.2*e* − 7 (0.8*e* − 7)	5.0*e* − 8 (0.6*e* − 8)	4.6*e* − 8 (0.5*e* − 8)

Residual						
*Ω*_*e*_	0.019 (0.0001)	0.290 (0.001)	0.003 (0.00001)	0.230 (0.001)	0.033 (0.0001)	0.033 (0.0001)

Deviance	−150487	217253	−398075	186380	−75029	−74306

Note: ln, natural logarithm; nu, normalized unit; NS, not significant. The statistically significant effects (Wald *Z*-test, *P* < 0.05), the fixed constant intercept *β*_0_, and sleep stage intercepts *β*_*s*_ are presented.

**Table 5:** Coefficients and their standard errors (SE) of the optimized multilevel model with the additional between-subject centering effect (Model #2) for the six cardiorespiratory parameters analyzed in this study.

Model coef.	Respiratory parameters		Cardiac parameters			
	BR, ln-Hz	SDBR, ln-Hz	HR, ln-bpm	SDNN, ln-ms	LF, nu	HF, nu
Fixed	Coefficient (SE)					
*β*_0_	−0.098 (0.079)^NS^	−0.012 (0.017)^NS^	0.104 (0.028)	−0.060 (0.047)^NS^	−0.018 (0.034)^NS^	0.131 (0.030)
*β*_*c*_	0.973 (0.011)	0.884 (0.020)	0.993 (0.007)	0.979 (0.011)	0.936 (0.012)	0.923 (0.011)
*β*_wake_	Baseline	Baseline	Baseline	Baseline	Baseline	Baseline
*β*_REM_	0.002 (0.008)^NS^	−0.199 (0.025)	−0.027 (0.004)	−0.104 (0.027)	0.030 (0.007)	−0.037 (0.007)
*β*_light_	−0.035 (0.008)	−0.606 (0.026)	−0.062 (0.004)	−0.052 (0.020)	−0.027 (0.005)	0.039 (0.006)
*β*_deep_	−0.044 (0.010)	−0.992 (0.033)	−0.054 (0.004)	−0.248 (0.026)	−0.096 (0.008)	0.105 (0.008)
*β*_*a*_		−0.002 (0.001)	−0.0001 (0.5*e* − 4)		0.0004 (0.0001)	0.0002 (0.0001)
*β*_*g*_	−0.024 (0.012)					
*β*_*b*_					0.005 (0.001)	−0.004 (0.001)
*β*_*t*_		0.0003 (0.0001)	−0.0001 (0.2*e* − 4)	0.001 (0.0001)	0.0004 (0.0001)	−0.0004 (0.0001)
*β*_*ta*_				−1.0*e* − 5 (0.1*e* − 5)		
*β*_*tg*_	0.0001 (0.5*e* − 4)					
*β*_*tb*_					−1.8*e* − 5 (0.5*e* − 5)	1.7*e* − 5 (0.5*e* − 5)

Random	Coefficient (SE)					
Ω_0_						
Ω_wake_	0.012 (0.001)	0.093 (0.011)	0.004 (0.0004)	0.094 (0.011)	0.006 (0.001)	0.005 (0.001)
Ω_REM_	0.014 (0.002)	0.099 (0.011)	0.003 (0.0003)	0.095 (0.011)	0.007 (0.001)	0.006 (0.001)
Ω_light_	0.006 (0.001)	0.061 (0.007)	0.002 (0.0003)	0.044 (0.005)	0.004 (0.001)	0.003 (0.0004)
Ω_deep_	0.010 (0.001)	0.131 (0.015)	0.003 (0.0003)	0.087 (0.010)	0.006 (0.001)	0.006 (0.001)
*Ω*_*t*_	1.1*e* − 7 (0.1*e* − 7)	6.7*e* − 7 (0.8*e* − 7)	3.5*e* − 8 (0.4*e* − 8)	7.1*e* − 7 (0.8*e* − 7)	4.8*e* − 8 (0.6*e* − 8)	4.3*e* − 8 (0.5*e* − 8)

Residual						
*Ω*_*e*_	0.019 (0.0001)	0.290 (0.001)	0.003 (0.00001)	0.230 (0.001)	0.033 (0.0001)	0.033 (0.0001)

Deviance	−151084	216873	−398866	185774	−75617	−74903

Note: ln, natural logarithm; nu, normalized unit; NS, not significant. The statistically significant effects (Wald *Z*-test, *P* < 0.05), the fixed constant intercept *β*_0_, and sleep stage intercepts *β*_*s*_ are presented.

**Table 6:** Variances of a sequence of models (Models A–G in the appendix) with different effects for computing their PVE for the six cardiorespiratory parameters analyzed in this study.

Models A–G with different effects (see the appendix)	Respiratory parameters		Cardiac parameters			
	BR, ln-Hz	SDBR, ln-Hz	HR, ln-bpm	SDNN, ln-ms	LF, nu	HF, nu
Model A: baseline model						
*Ω*_*e*_	0.0229	0.3306	0.0043	0.2626	0.0354	0.0356
*Ω*_0_	0.0328	0.1389	0.0192	0.2997	0.0151	0.0156
Dev	−125045	232926	−348717	202249	−66487	−65952

Model B: Model A + within-subject time effect (fixed)						
*Ω*_*e*_	0.0228	0.3284	0.0040	0.2600	0.0353	0.0355
*Ω*_0_	0.0328	0.1393	0.0191	0.2999	0.0150	0.0155
Dev	−125109	232056	−357783	200926	−66724	−66131

Model C: Model B + demographic effect (fixed)						
*Ω*_*e*_	0.0228	0.3284	0.0040	0.2600	0.0353	0.0355
*Ω*_0_	0.0308	0.1329	0.0183	0.2230	0.0147	0.0136
Dev	−125120	232048	−357790	200877	−66730	−66152

Model D: Model C + centering effect (fixed)						
*Ω*_*e*_	0.0228	0.3284	0.0040	0.2600	0.0353	0.0355
*Ω*_0_	0.0001	0.0098	0.0001	0.0033	0.0003	0.0002
Dev	−126064	231624	−358718	200200	−67367	−66850

Model E: Model D + demographic-related time effect (fixed)						
*Ω*_*e*_	0.0227	0.3284	0.0040	0.2597	0.0352	0.0354
*Ω*_0_	0.0001	0.0098	0.0001	0.0033	0.0003	0.0002
Dev	−126393	231624^Ne^	−358718^Ne^	200027	−67718	−67206

Model F: Model E + between-subject time effect (random)						
*Ω*_*e*_	0.0210	0.3157	0.0034	0.2476	0.0343	0.0346
*Ω*_0_	0.0003	0.0097	0.0001	0.0041	0.0003	0.0002
*Ω*_*t*_	1.1*e* − 7	7.3*e* − 7	3.6*e* − 8	7.1*e* − 7	5.2*e* − 8	4.5*e* − 8
Dev	−136185	226933	−380964	194316	−70913	−69899

Model G: Model F + between-subject physiological effect (random)						
*Ω*_*e*_	0.0186	0.2896	0.0029	0.2298	0.0328	0.033
*Ω*_0_	0	0	0	0	0	0
*Ω*_*t*_	1.0*e* − 7	6.7*e* − 7	3.5*e* − 8	7.1*e* − 7	4.8*e* − 8	4.3*e* − 8
Dev	−151084	216874	−398866	185774	−75617	−74903

Note: ln, natural logarithm; nu, normalized unit; Dev, model deviance; Ne, no effect. All the models include fixed (*β*_0_) and random (*μ*_0_) intercepts, and sleep-stage-dependent variables (*wake*, *REM*, *light*, and *deep*) with their coefficients. The models were optimized by excluding the effects with their coefficients statistically equal to zero (Wald *Z*-test, *P* > 0.05) and the variances presented in the table were all statistically significant (Wald *Z*-test, *P* < 0.01).

**Table 7:** Proportion of variance explained (PVE, %) accounted for by different effects for the six cardiorespiratory parameters analyzed in this study.

Effect	Respiratory parameters		Cardiac parameters			
	BR	SDBR	HR	SDNN	LF	HF
Overall between-subject effect						
Demographic effect	3.55%	1.37%	3.36%	13.69%	0.63%	3.70%
Centering (physiological) effect	55.26%	26.23%	77.95%	39.06%	28.63%	26.41%
Between-subject time effect	2.74%	2.72%	2.67%	2.00%	1.87%	1.58%
Between-subject physiological effect	5.03%	7.62%	2.27%	3.91%	3.49%	3.44%
Overall within-subject effect						
Within-subject time effect	0.01%	0.37%	1.32%	0.42%	0.16%	0.14%
Within-subject physiological effect	33.39%	61.69%	12.43%	40.87%	65.04%	64.54%
Cross-interaction effect						
Demographic-related time effect	0.02%	Ne	Ne	0.06%	0.18%	0.19%

Note: ln, natural logarithm; Ne, no effect. For each cardiorespiratory parameter, the sum of PVEs from all the effects is 100%, representing the total variance for that parameter. The centering effect reflected some between-subject physiological variability (at the overnight mean level) that was assumed to be independent of sleep stage composition over the entire night.

**Table 8:** Comparison of sleep staging results (wake/REM sleep/light sleep/deep sleep) using different schemes in correcting the cardiorespiratory parameters.

	PSG	BS	CS1	CS2	CS3
Overall performance					
Accuracy, %	—	55.8 ± 9.8	60.4 ± 8.8	62.9 ± 7.8	83.5 ± 14.4
Kappa coefficient	—	0.19 ± 0.10	0.29 ± 0.11	0.35 ± 0.09	0.72 ± 0.23
Sleep stage composition (percentage)					
Wake, %	19.8 ± 12.5	19.9 ± 14.4	18.4 ± 4.9	20.6 ± 6.4	19.7 ± 10.7
REM sleep, %	14.0 ± 5.6	0.7 ± 1.0	2.4 ± 2.0	3.0 ± 1.7	10.5 ± 7.8
Light sleep, %	53.4 ± 10.7	74.7 ± 15.1	73.5 ± 8.1	71.0 ± 8.2	59.9 ± 12.0
Deep sleep, %	12.8 ± 7.2	4.7 ± 5.6	5.7 ± 5.2	5.4 ± 4.0	9.9 ± 7.6

Note: BS, baseline with original parameter values without correction; CS1, with correction by fixed effects; CS2, with correction by fixed effects and between-subject random effects; CS3, with correction by fixed effects and within-subject random effect (model residual). For CS2 and CS3, results were obtained when assuming the sleep stages were known, which was usually not the case in practice. For accuracy and Kappa coefficient, significance of difference between using each correction scheme and BS was confirmed with a paired (two-sided) Wilcoxon signed-rank test, all at *P* < 0.00001.

## Appendix

The sequence of models constructed to compute the PVE values for different effects is described in as follows.

(i) The first model is the model with solely the constant and random intercepts as well as the fixed sleep stage dependent variables. This baseline model can be written as

Model A:
  (A.1) yij=β0A+μ0jA+∑sβsAsij+e0ijA,with  μ0jA~N0,Ω0A,  e0ijA~N0,ΩeA,

where *s* = wake, REM, light, and deep sleep and the total variance *Ω*_total_ consists of variance in two levels: the between-subject variance *Ω*_0_^A^ at the subject level and the within-subject (residual) variance *Ω*_*e*_^A^ at the time/epoch level. The percentage of the total variance taken by *Ω*_0_^A^, called intragroup correlation coefficient (ICC) *ρ* [43, 59], is computed by

(A.2) $$\begin{array}{c} \rho =\frac{\Omega_{0}^{A}}{\Omega_{total}}=\frac{\Omega_{0}^{A}}{\left(\Omega_{e}^{A}+\Omega_{0}^{A}\right)}. \end{array}$$

(ii) Let us then consider the model with fixed time effect at the first level

Model B:
  (A.3) yij=β0B+μ0jB+∑sβsBsij+βtBtimeij+e0ijB,with  μ0jB~N0,Ω0B,  e0ijB~N0,ΩeB.

For the variance analysis of the time variable, instead of using the original time stamps mentioned before (i.e., time_*ij*_ = *i*/2), we use the shifted (centered) values computed as the original time minus the mean value of the median time over all subjects. This is because, for a longitudinal multilevel analysis, time is an occasional variable within subjects and it usually suffices a linear trend for the measurements since it thus would explain part of total variance in both levels [43]. Actually, with and without shifting the occasion measures do result in equivalent models with exactly the same model coefficients (including residual) and deviance except for the variance estimates of random effects. The variance estimates obtained by shifting the time values are considered to be more accurate and realistic [43]. To quantify the PVE constituted by the fixed time effect, we exploit the relative variance reduction of the baseline model in the two levels *R*_1_^2^ and *R*_2_^2^, such that

(A.4) PVEtime_fixed1−ρR12+ρR22=ρΩeA−ΩeBΩeA+1−ρΩ0A−Ω0BΩ0A=ΩeA−ΩeB+Ω0A−Ω0BΩtotal.

Now we consider the subject-level fixed effects.

(iii) The model including demographic variables is as follows:

Model C:
  (A.5) yij=β0C+μ0jC+∑sβsCsij+βtCtimeij+e0ijC+βaCagej+βgCgenderj+βbCBMIjwith  μ0jC~N0,Ω0C,  e0ijC~N0,ΩeC.

Similarly, the PVE explained by the between-subject demographic variables can be computed by

(A.6) $$\begin{array}{c} {\text{PVE}}_{\text{demographic}}=\frac{\left[\left(\Omega_{e}^{B}-\Omega_{e}^{C}\right)+\left(\Omega_{0}^{B}-\Omega_{0}^{C}\right)\right]}{\Omega_{\text{total}}}. \end{array}$$

The demographic variables only explain the variability between subjects, so the variance change at the epoch level should be approximately zero (*Ω*_*e*_^B^ − *Ω*_*e*_^C^ = 0).

(iv) Further, Model D is the model with the inclusion of between-subject centering effect (expressing the physiological difference between subjects at the overnight mean level), given by

Model D:
  (A.7) yij=β0D+μ0jD+∑sβsDsij+βtDtimeij+βcDy−j+e0ijD+βaDagej+βgDgenderj+βbDBMIjwith  μ0jD~N0,Ω0D,  e0ijD~N0,ΩeD,

from which the corresponding PVE is computed such that

(A.8) $$\begin{array}{c} {\text{PVE}}_{\text{center}}=\frac{\left[\left(\Omega_{e}^{C}-\Omega_{e}^{D}\right)+\left(\Omega_{0}^{C}-\Omega_{0}^{D}\right)\right]}{\Omega_{\text{total}}}. \end{array}$$

(v) For the inclusion with cross-interactions that express the demographic-related time effects, the model is as follows:

Model E:
  (A.9) yij=β0E+μ0jE+∑sβsEsij+βtEtimeij+βcEy−j+e0ijE+βaEagej+βgEgenderj+βbEBMIj+βtaEtime×ageij+βtgEtime×genderij+βtbEtime×BMIij,with  μ0jE~N0,Ω0E,  e0ijE~N0,ΩeE,

and the proportion of cross-interaction variance is

(A.10) $$\begin{array}{c} {\text{PVE}}_{\text{cross}}=\frac{\left[\left(\Omega_{e}^{D}-\Omega_{e}^{E}\right)+\left(\Omega_{0}^{D}-\Omega_{0}^{E}\right)\right]}{\Omega_{total}}. \end{array}$$

In addition to the fixed part, we consider the random part of some effects.

(vi) The model with additional random time effect is as follows:

Model F:
  (A.11) yij=β0F+μ0jF+∑sβsFsij+βtF+μtjFtimeij+βcFy−j+e0ijF+βaFagej+βgFgenderj+βbFBMIj+βtaFtime×ageij+βtgFtime×genderij+βtbFtime×BMIij,with  μ0jFμtjF~N00,Ω0FΩtF,  e0ijF~N0,ΩeF.

The computation of the PVE accounted for by the random time effect can be accordingly obtained by

(A.12) $$\begin{array}{c} {\text{PVE}}_{\text{time\_random}}=\frac{\left[\left(\Omega_{e}^{E}-\Omega_{e}^{F}\right)+\left(\Omega_{0}^{E}-\Omega_{0}^{F}\right)\right]}{\Omega_{total}}. \end{array}$$

(vii) Afterwards, the model with random effects for different sleep stages (expressing the between-subject physiological variability associated with each sleep stage in random part) is then expressed as

Model G:
  (A.13) yij=β0G+μ0jG+∑sβsG+μsjGsij+βtG+μtjGtimeij+βcGy−j+e0ijG+βaGagej+βgGgenderj+βbGBMIj+βtaGtime×ageij+βtgGtime×genderij+βtbGtime×BMIij,with  μ0jGμtjGμsjG~N000,Ω0GΩtGΩsG,  e0ijG~N0,ΩeG.

In this model, the random variance *Ω*_*s*_^G^ not only explains the variance in *Ω*_0_^F^ and *Ω*_*e*_^F^, but also reflects some variance of the random time effect *Ω*_*t*_^F^. Therefore, the proportion of variance contained in *Ω*_*s*_^G^ to the total variance is as follows:

(A.14) PVEbetw_subj_random=ΩeF−ΩeG+Ω0F−Ω0G+ΩtF−ΩtGΩtotal.

Then the PVE of the random time effect to the total variance should be corrected to

(A.15) PVEtime_random=ΩeE−ΩeF+Ω0E−Ω0F−ΩtF−ΩtGΩtotal.

(viii) Finally, the remaining residual variance is assumed to only associate with the physiological variability within subjects and its proportion can be obtained such that

(A.16) $$\begin{array}{c} {\text{PVE}}_{\text{within\_subj\_random}}=\frac{\Omega_{e}^{G}}{\Omega_{\text{total}}}. \end{array}$$

Note that all these models are optimized by only keeping the variables that do not statistically equal zero.
